# South West Orthopoedic Club, Meeting at Taunton, November 1987

**Published:** 1989-02

**Authors:** 


### South West Orthopaedic Club

### Meeting at Musgrove Park Hospital, Taunton on November 7th, 1987

### EARLY EXPERIENCE WITH GORETEX PROSTHETIC CRUCIATE LIGAMENT RECONSTRUCTION

**Philip V Seal and I S R Reynolds, Hereford**

*Text*: Cruciate ligament rupture, anterior in the main, is a common injury. The management of significantly disabled patients continues to present a complex and debatable problem. Prosthetic ligaments have facilitated management but long term results are awaited.

This paper analyses the first 35 of 44 patients treated with Goretex prosthetic cruciate ligament reconstruction studied prospectively for up to $2\frac{1}{4}$ years. Thirty three patients had ACL rupture, one PCL rupture and one rupture of both ligaments.

*Results*: The majority (31) of the patients were male, in the third decade (54%) and were keen on recreational sport. The major sports were soccer, squash and rugby. The predominant mechanism of injury was a twist in 20 patients and a valgus injury in 9. Nine patients heard and felt a snap, only 5 patients had the diagnosis made at the time of presentation. Associated injuries were eight torn medial menisci, one torn lateral miniscus, four torn medial collateral ligaments and ten patients had articular cartilage damage to the medial femoral condyle.

The predominant symptom was giving way at sport in 29 patients and giving way with sedentary activity in 16. Locking occurred in 5, pain in 1 and swelling in 14. There was no direct correlation between laxity demonstrated by the anterior drawer at 90°, the Lachman test nor the pivot shift with the patients instability. Under anaesthesia, the anterior drawer was more significant than the Lachman or pivot shift tests. Operative problems were minimal. Notch-plasties were undertaken on 15 occasions. The majority of patients were discharged from hospital in less than 7 days. At the completion of surgery, the anterior drawer was absent in 30, Lachman in 33 and pivot shift in all 35. Minimal anterior drawer and Lachman were noted in the others.

Immediate post-operative complications were minimal. Late sterile effusion occurred in 8 patients, in two on three occasions. These occurred from four months onwards, all resolved satisfactorily and were usually assocated with vigorous activity or a further injury. Turbid, pale, sterile fluid was aspirated revealing the presence of histiocytes. Two prostheses have ruptured when the patients returned to playing rugby, both have been revised. Follow up is for a maximum of $2\frac{1}{4}$ years. Eighteen patients have returned to their sport and 27 have regained full knee movement. Seventeen patients were symptom free, 18 had a little pain, 3 the sensation of insecurity and 7 were apprehensive concerning returning to sport. Subjectively, 28 considered the result excellent, 4 satisfactory and 3 poor. Considerable improvement in the demonstrable laxity was evident. Some increase in the anterior drawer and Lachman does occur with time.

*Conclusion*: Goretex prosthetic reconstruction of ruptured cruciate ligaments seems in the short term to be a satisfactory mode of treatment for significantly disabled patients. Long term results are awaited.

We have reservations about patients returning to high class rugby. Chronic synovitis has not yet occurred but recurrent sterile effusion is a problem. Our early results encourage us to continue this mode of treatment.

### EARLY OSTEOARTHRITIS IN YOUNG SPORTSMAN WITH SEVERE ANTEROLATERAL INSTABILITY OF THE KNEE

**G Graham and J A Fairclough, Cardiff**

In a retrospective study of 240 patients presenting with symptoms of knee instability; 16 were discovered who had developed severe chondromalacia or osteoarthritis of the femoral condyles. All of the patients gave a history of a previous severe knee injury occurring in their teenage years and all had continued to play competitive sport. Ten of the group had subsequent meniscal injuries requiring surgery.

In 9 of the group, previous arthroscopic or open joint procedures had demonstrated normal femoral joint cartilage. There was no difference in the degree of degeneration in those who had had a meniscectomy as compared to those who had not had meniscal damage.

Evidence is presented that early radiological changes representing degeneration of the knee are present following a rupture of the anterior cruciate ligament.

It is concluded that severe antero-lateral instability is a cause of early degenerative joint disease in young athletes even in the absence of meniscal damage. The role of anterior cruciate reconstruction was discussed.

### THE INCIDENCE AND EFFECT OF WOUND INFECTION IN ELECTIVE ORTHOPAEDIC SURGERY

**Gordon Bannister, Southmead Hospital, Bristol**

The risks of infection in prosthetic arthroplasty are well recognized, but those in elective orthopaedic cases which do not involve implants have tended to be overlooked.

Over a 6 year period from 1981–1987 the perioperative infections recorded in the Sepsis Report of Winford Orthopaedic Hospital were related to the number of cases performed during this time.

There were 182 infections from 12,032 consecutive open procedures (1.51%). Coagulase positive staphylococci alone accounted for 32% of all infections and coagulase negative staphylococci for 17%. Staphylococci were associated with 66% of mixed infections. The proportion of infected organisms over the 6 years of the study did not change. Infection rates varied according to the operation performed. High risk procedures were revision total knee replacement, arthrodesis following knee replacement, ankle and subtalar hindfoot fusion and spinal fusion. 15% of revision knee replacements incurred a perioperative infection. Hindfoot and spinal fusion carried infection rates of 7%. The morbidity of these procedures as a consequence of sepsis was increased by 28 and 9

weeks respectively. The pseudoarthrosis rate was between 20 and 25%, when infection had taken place.

Infection did not vary according to the theatre in which the procedures were carried out, but did vary by year. The years in which infection was significantly higher were those in which building and construction work had taken place within the theatre complex.

Prophylactic antibiotics are already used for prosthetic joint replacement. There will be an 8% increase in their use if they were employed for hindfoot and spinal fusions. This could bring a welcomed reduction in morbidity, whilst limiting the financial implications and effects on bacterial ecology.

### THREE DOSE SYSTEMIC ANTIBIOTIC OR BONE CEMENT INCLUDING ANTIBIOTIC IN THE PROPHYLAXIS OF INFECTION AFTER TOTAL JOINT REPLACMENT

**P C May, Bath and Wessex Orthopaedic Hospital, Bath**

This paper reports the incidence of infection in a prospective, randomised, multicentric trial of 435 patients undergoing major joint arthroplasty protected by antibiotic administered peri-operatively by one of two routes. The rates of infections in this series differed little between the two groups.

The trial period commenced in January 1982 and patients were entered until December 1985. Briefly at induction for surgery, patients were randomly allocated to either three doses iv systemic Cephuroxine (Zinacef) or to a similar quantity of Cephuroxine divided and mixed dry into the bone cement before use at operation. All entered patients were followed up for two post-operative years in one of the three orthopaedic centres involved. Record cards have not only listed pre-operative diagnosis, arthroplasty type and operative approaches (these will be discussed) but included careful records of post-operative wound and intercurrent infections which we presented and analysed.

After exclusions, there remained 204 patients in each group in each of the two groups. The trial definitions of Superficial or Deep and Early or Late infections will be explained. Early wound infections were seen in 5.4% of cases in the patenteral group and 9.3% of the cement group. There was one Deep early infection in the patenteral group, 0.5% and two in the cement group, 0.9%. Statistically, no difference could be shown. Five out of the 28 cases of Early superficial infection progressed to Late infections. Within this 18%, no statistical differences were apparent. There is no evidence to suggest that chest or other infections post-operatively predisposed these patients to late wound infections.

Late, deep infections were diagnosed in four patients in the parenteral group, 1.9% and in three patients in the cement group, 1.4%. Not all cases have been surgically explored at this time. Statistically, there is no significant difference in the incidence of late, deep infection between these two cohorts.

Both techniques of administration appear to give comparable protection in the prophylaxis of infection: Superficial or Deep, Early or Late (up to 2 years). The low incidence of infection in each group is comparable with other published series.

### PELVIC INJURIES ASSOCIATED WITH TRAUMATIC ABDUCTION OF THE LEG

**D J A Scott and J D Bear, Vascular Studies Unit, Bristol Royal Infirmary, Bristol**

Open fractures of the pelvis are rare but when they do occur the severity of the associated injuries is often underestimated and consequently the morbidity and mortality due to haemorrhage and sepsis may exceed 50%.

We report a series of 5 road traffic accidents (4 motor cyclists), 4 men and 1 woman aged 20–31 years. All sustained a severe fracture of the pelvis associated with a deep perineal laceration caused by forced abduction of the leg. All patients were shocked on arrival in casualty, clinically both the symphysis pubis and sacroiliac joints were disrupted. Resuscitation with colloid and blood failed to maintain an adequate blood pressure and only then was the possibility of an intra-abdominal injury considered.

One patient died shortly after arrive in casualty. Examination under general anaesthesia revealed the full extent of the perineal laceration which extended from the groin to the anal margin in all cases. The anal sphincters were damaged with disruption of the pelvic floor but the rectum remained intact. Three patients had compound fractures of the femur on the side of the disrupted hemipelvis. Packing of the perineal laceration failed to control haemorrhage in 3 cases necessitating laparotomy which revealed an extensive pelvic haematoma extending into the retroperitoneum. In one patient the blood pressure could only be restored by cross clamping the infrarenal aorta and in two cases ligation of the internal iliac arteries failed to control the haemorrhage. Packing of the pelvis alone failed to control haemorrhage because of gross instability of the hemipelvis. External fixation in combination with pelvic packs in 2 cases and 1 without packs resulted in haemostasis. A pelvic sling was used in the remaining case but there was persistent bleeding and subsequently the patient died from a consumptive coagulopathy. The transfusion requirements ranged from 10–58 units.

These cases identify the need for a multidisciplinary approach to open pelvic fractures. If both morbidity and mortality are to be reduced coagulopathy and sepsis must be avoided at an early stage. The application of a G suit may reduce the initial blood loss; cross clamping the infrarenal aorta allows early operative control but ligation of the internal iliac arteries is often ineffective because of the venous injuries. For packing to be effective the associated pelvic fracture must be stabilised by external fixation. Disruption of the anal sphincters is common and best treated by a sigmoid loop colostomy and a delayed anal repair.

### SETTING UP A C.D.H. SCREENING PROGRAMME

**T P Green, Gloucestershire Royal Hospital**

In 1985 the screening procedure for congenital dislocation of the hip was reviewed in the Gloucester Health Authority (annual average birth rate—3,000).

It was found that despite a heavy orthopaedic input, and ninety children a year being treated in a Craig splint, five cases were missed and presented late.

A new protocol was commenced in which more emphasis was placed on a positive family history and other risk factors such as breech presentation or moulded feet, in order to select which children were followed up in addition to those found to be symptomatic at birth.

After the new programme had been running for over a year the results were compared with a similar period under the old system.

Two children have so far been picked up under the new arrangements with a true congenital dislocation, which would have been missed under the old system. Both had been normal at birth but had been selected for follow up because of a positive risk factor.

The new screening programme, although in its infancy, does seem to provide a more logical and thorough screening of children likely to be at risk.

The worries associated with such a programme include the risk of introgenically induced hip disorder, prompting the development of non-manipulative screening methods. There is also the possibility of an increasing percentage of births falling into a family history risk group if this is too loosely defined.

*Continued on page 32a.*

**2. South West Orthopaedic Club**

*Continued from page 28.*

### METASTATIC DISEASE OF THE THORACIC & LUMBAR SPINE

**D A P Ainscow, Cheltenham General Hospital, Cheltenham**

The changing approach of orthopaedic surgeons to this condition was described. The recognition that two types of metastatic disease occur, one in the vertebral body which can be treated successfully surgically by anterior surgery and a second paracaudal metastatic disease which is treated less successfully by surgery but in which posterior procedures may be of benefit.

Successful anterior surgical treatment requires good decompression of the spinal canal. Various methods of stabilisation may be used depending on the life expectancy of the patient and the degree of instability. Mention was made of the usefulness of the vascularised rib graft. Problems of diagnosis and patient selection were discussed. The results of nine cases of metastatic disease, six treated anteriorly and three posteriorly, were presented. There were two deaths within the first eight days but no patient deteriorated neurologically during surgery and the relief of pain was the most vuluable benefit. Patients with solitary spinal deposits from unknown primaries are a particularly satisfying group to treat.

